# The Enhanced Mentor Mother ProgrAm (EMMA) for the prevention of mother-to-child transmission of HIV in Kenya: study protocol for a cluster randomized controlled trial

**DOI:** 10.1186/s13063-018-2975-y

**Published:** 2018-10-30

**Authors:** Bruce A. Larson, Margaret Bii, Isaac Tsikhutsu, Nafisa Halim, Vanessa Wolfman, Peter Coakley, William Sugut, Fredrick Sawe

**Affiliations:** 1 Department of Global Health, Boston University School of Public Health, Boston, MA USA; 2 Kenya Medical Research Institute/Walter Reed Project, Kericho, Kenya; 3 HJF Medical Research International, Inc., Nairobi, Kenya; 4 US Military HIV Research Program (MHRP), Henry Jackson Foundation, Bethesda, MD USA

**Keywords:** AIDS, Antiretroviral therapy, Prevention of mother to child transmission, Mentor mothers, EMMA, Kenya

## Abstract

**Background:**

As of September 2014, Kenya implemented the WHO recommended Option B+ guidelines in which all newly diagnosed HIV-infected pregnant women are immediately eligible for triple antiretroviral therapy (ART) for life regardless of CD4 count. In addition, Kenya previously established the Kenya Mentor Mother Program (KMMP) in 2012 to improve peer education and psychosocial support services within the national prevention of mother-to-child transmission (PMTCT) program. The primary objectives of the study described in the current protocol are: (1) to evaluate implementation of these new guidelines (Option B+ with Mentor Mothers) as part of routine service delivery; and (2) to evaluate potential benefits of a package of services within the KMMP (called EMMA) to improve PMTCT service delivery.

**Methods:**

We will conduct a cluster randomized controlled trial in western Kenya. We will allocate 12 clinics providing PMTCT services including ART to two study arms using pair matching: the standard of care (SOC) arm, which includes the KMMP as implemented by the clinics; and the intervention arm, which is the SOC (including KMMP) with the EMMA package of services (a targeted exit interview, visit reminders, and targeted follow-up). At the intervention clinics, the EMMA package of services is implemented as part of routine service delivery. A total of 360 (180 in each arm) pregnant women will be enrolled in the study at or near their first visit for antenatal care for prospective records review through 72 weeks post-partum. The primary and secondary outcomes are uninterrupted supplies of ART medications throughout the PMTCT cascade of care as well as infants completing HIV testing on schedule.

**Discussion:**

The EMMA package of services provides specific structure to the use of Mentor Mothers within PMTCT programs. This strategy was developed in collaboration with local health facility and PMTCT program staff based on their experience providing PMTCT services within the integrated ART-MCH facilities. If successful, this approach has the potential to improve dramatically PMTCT service delivery with minor additional costs beyond the basic mother–mentor program and support global goals to eliminate mother-to-child transmission.

**Trial registration:**

ClinicalTrials.gov, NCT02848235. Registered on 19 July 2016.

**Electronic supplementary material:**

The online version of this article (10.1186/s13063-018-2975-y) contains supplementary material, which is available to authorized users.

## Background

### Background and rationale

In Kenya, HIV-infected pregnant women should receive prevention of mother-to-child transmission (PMTCT) services, beginning with their first visit for antenatal care and lasting through 18 months after delivery (or cessation of breastfeeding with could occur later than 18 months). As of 2014, the Kenyan Ministry of Health recommended “Option B+” to be included into the standard PMTCT package of care, which includes the “immediate initiation of life-long ART in pregnant and breastfeeding women upon HIV diagnosis with continuous adherence support” (p. 16, [1]). The 2014 recommendations also concluded that “deliberate and focused patient support and defaulter tracking mechanism need to be put in place to ensure compliance to HIV treatment” (p. 16, [1]). As of September 2016, the Kenyan Ministry of Health reaffirmed its commitment to Option B+ and expanded the frequency of HIV testing for HIV-exposed infants (infants born to HIV-positive mothers) to 6, 24, 48, and 72 weeks [2].

Before 2016 in Kenya, treatment guidelines have been very specific on when to initiate antiretroviral therapy (ART) and what to initiate (which medications). Guidelines have been less explicit on where women should initiate and be managed on life-long ART during pregnancy and breastfeeding. Recognizing the barriers pregnant women newly diagnosed with HIV during antenatal care would face if they were referred elsewhere for HIV care and treatment, in 2005 the Kenyan South Rift Valley PEPFAR (SRVP) program began to integrate all HIV-related care, including ART, into PMTCT services provided at the larger maternal and child health clinics within the region. This was an early and innovative approach that is consistent with newer ideas for differentiated service delivery (DSD) or differentiated models of care (DMC) [3]. As of 2013, when the study described in the current protocol was being developed, this approach was implemented in the largest facilities providing PMTCT services in the SRVP.

As part of the standard of care (SOC) beginning in 2012, Kenya also developed the Kenya Mentor Mother Program (KMMP) [4], which follows on similar approaches implemented in other countries (e.g. see [5–7]). Mentor Mothers are clinic staff members who are also women living with HIV. They are trained and employed to be part of the clinic’s medical team to provide pre-test group education sessions, one-on-one and couples counseling, support groups, and defaulter tracking. As part of their routine clinic activities, Mentor Mothers obtain consent from their clients for various forms of follow-up, including consent for both phone and SMS follow-up (or only phone follow-up or no follow-up) as well as consent for home follow-up by her or a community health worker.

Information continues to be lacking in Kenya and elsewhere on the ability of PMTCT programs to initiate pregnant women with HIV on ART during pregnancy and retain them on treatment along the PMTCT cascade of care through delivery, cessation of breastfeeding, and for the longer term. What information exists suggests significant room for improvement. For example, a recently published study from a treatment program in western Kenya, with an active outreach department, reported that 32% of women initiated on ART during pregnancy disengaged from treatment before delivery [8]. Although the data for this study were from 2006 to 2009, they indicate the difficulties with retaining pregnant women on ART during pregnancy. Information on other key outcomes, such as the proportion of those eligible who actually initiated treatment are not reported. Another study from roughly the same region of Kenya during the same time period reported that 38% of women newly diagnosed with HIV during pregnancy did not register in the PMTCT program [9]. In two hospitals between Kericho and Nairobi (Gigil and Naivasha), only 4% of women eligible for ART initiated treatment within six months of their HIV diagnosis [10].

Some evidence suggests that integration of ART within maternal and child health (MCH) clinics, such as exists within the study region, improves linkage to and initiation of treatment. For example, a study from Zambia found that integration of PMTCT services including ART into MCH services increased the percentage of women who initiated PMTCT care (44% compared to 25%) and increased the percentage of those eligible who initiated treatment during pregnancy (32% compared to 4%) [11]. While integration improved upon these basic outcomes, the majority of patients still did not initiate PMTCT care or initiate ART if eligible. Early evidence on the implementation of life-long ART for pregnant women from Malawi suggests that eliminating CD4 counts as an eligibility criterion might facilitate initiation, but some significant share may still not agree to initiate ART and retention on treatment during and after delivery may remain problematic without specific attention directed towards these issues [12].

To support effective implementation of the Kenya PMTCT SOC in the South Rift Valley and other regions of Kenya, better and up-to-date information on implementation is still needed. Recognizing the importance of both timely initiation and retention on treatment during and after pregnancy, active strategies for retaining patients in care are preferable to after-the-fact follow-up to re-engage a patient in care who previously disengaged. Thus, feasible programmatic interventions can support successfully implementation of the SOC and long-term patient care beyond pregnancy.

The study described in the current protocol is designed to fill this information gap and to improve implementation of the Kenyan PMTCT SOC (which includes Option B+ and Mentor Mothers). We will evaluate the impacts of an innovative strategy to improve implementation of the SOC. The strategy, called the *E*nhanced *M*entor *M*other Progr*A*m (EMMA), provides specific structure to the use of Mentor Mothers within PMTCT programs. If successful, this approach has the potential to improve PMTCT service delivery and support global goals to eliminate mother-to-child transmission. This enhancement to the existing Mentor Mother program is consistent with the KMMP guidelines, which emphasizes that “the National Guidelines do not intend to remove the space for creativity and innovation in these approaches. On the contrary, the MOH hopes to inspire ongoing dialogue about quality improvements from the clearly defined starting point outlined herein” [4].

### Trial design

We will conduct a parallel, two-arm, cluster randomized controlled trial to examine the effect of the EMMA strategy on retention in the PMTCT cascade of care among HIV-positive pregnant and postpartum women, as well as HIV-exposed infants completing HIV testing on schedule. In addition, the cost to clinics of organizing and managing the revised Mentor Mother daily activities to provide EMMA services will also be assessed. A cluster randomized design is required as enhanced service delivery is implemented at a site level rather than at an individual patient level (see, e.g. [13] for additional discussion of study designs for quality improvement evaluations). The study will enroll patients in both study arms for prospective review of medical records and other information collected by the sites as part of routine patient care.

This study protocol follows the Standard Protocol Items: Recommendations for Interventional Trials (SPIRIT) guidelines (see Additional file 1 – SPIRIT Checklist).

### Description of the EMMA intervention

Before providing additional information on the study design and methods, it is useful to provide additional detail on the EMMA intervention first. Sites randomized to the SOC arm will continue to implement the Kenyan PMTCT SOC for all patients. Sites randomized to the intervention arm will implement EMMA as an addition to their SOC during the study period for all patients. Each pregnant woman with HIV presenting for antenatal care will receive the site’s SOC (including the KMMP) with the following additions:

1. Exit discussion with the Mentor Mother

At the end of each MCH clinic visit, from the first ANC visit through 18 months post-partum, all women with HIV will be directed to a Mentor Mother**.** For perspective, sites typically have 1–2 Mentor Mothers. In the KMMP, the SOC does not specify the frequency at which one-on-one counseling should occur, only that at least one should occur over the course of a patient’s pregnancy. With EMMA, the Mentor Mother will complete an exit discussion/counseling session with the patient, at the end of each visit, to review clinical care received at the visit, answer questions, discuss any concerns of the patient, review the schedule for her next visit, and discuss the importance of attending the next visit (see Additional file 2 – Mentor Mother Guide).

2. Reminder messages

At each exit discussion, the Mentor Mother will give each woman the option to receive an automatic text message from the Mentor Mother’s smartphone (Android) before her next visit (on a day and time of her choosing) to assist with planning for the visit. The message will say, “This is your reminder for your MCH visit on ‘DAY’, ‘DATE’”. This message option will be offered to women at each encounter because visit schedules are not standard for all patients and vary by gestational age, time after delivery, mothers’ and/or babies’ health, etc. The patient can opt to receive a text message at some visits but not others. If the patient does not have a cell phone, she can have a message sent to any other phone she would prefer. The Mentor Mother will also offer to set up a reminder directly in the patient’s cell phone on the date and time she would like the reminder to show on her phone (acts like a text message in an alarm). The Mentor Mother will also set up an automatic reminder on her cell phone for the day a patient is expected to return. In Kenya, the recipient of a text message incurs no cost. Text messages will be sent using SMS Scheduler, which is a free Android-based application that is installed on a study-purchased Smart phone provided to the Mentor Mother. This app was preferred by the study team due to its ability to store message delivery status and dates, which can be accessed periodically by the study team to document the reminders sent to each patient.

3. Patient follow-up

If the patient does not return to the clinic at a scheduled visit, the Mentor Mother will wait for an additional day to see if the patient returns. If she does not, she will send a text to the patient and call the patient to follow-up (and counsel to return).

The EMMA intervention was developed in collaboration with local health facility and PMTCT program staff based on their experience providing PMTCT services within the integrated ART-MCH facilities. The intervention was designed to be: (1) feasible for sites to implement as an addition to routine practices; (2) already consistent with policies for patient management, interactions, and follow-up in Kenya; and (3) affordable for sites to adopt as routine practice. In short, this is an implementation science study that addresses how to improve implementation of an already know effective intervention, ART [14].

A number of other approaches were explored to optimize implementation of the 2016 SOC, such as subsidizing transportation costs, various incentives for completing visits on schedule (e.g. for six-week infant HIV testing), or automatic text messages for multiple future visits that could be set up without the Mentor Mother interaction with the patient. After reviewing such options in detail with program and site staff, such approaches were not considered feasible for sites to implement in the future as part of routine practice.

### Objective

The objective of this study is to assess adherence to scheduled clinic visits, retention in care, and infant HIV testing along key segments of the PMTCT cascade and the effect of EMMA on retention from study enrollment at or near the first visit for antenatal care through 18 months post-partum. In addition, the study will estimate the additional cost to the PMTCT program of implementing the EMMA strategy.

## Methods

### Study setting

We selected 12 health facilities providing integrated MCH and ART services in the study region as part of the Kenya Medical Research Institute (KEMRI)/Walter Reed Project (WRP) SRVP. These facilities provide the majority of PMTCT services for HIV-infected pregnant and post-partum women in the region. This region is ideal for this study for several reasons, including: all study sites are implementing the 2016 SOC; each site is a major MCH clinic providing PMTCT services in the region; ART services are already integrated into MCH services in all sites; the sites are geographically distributed across the region in Kisumu, Siaya, Kericho, Nandi, Bomet, and Narok Counties; and strong support in the region exists for study (from County Governments, KEMRI/WRP Community Advisory Board, and sites).

### Study population

The study population comprises all pregnant women with HIV presenting at a study site for their first antenatal care (ANC) visit. This includes women who are newly diagnosed with HIV as part of their first ANC visit (which is routine practice in the region and study sites) as well as pregnant women with HIV who are already on ART at their first visit for antenatal care.

#### Inclusion criteria

- Adult patients (aged 18 years or older);
- Pregnant women with HIV presenting for antenatal care at a study site;
- The ability to understand and the willingness to sign/mark a written informed consent document in English, Kiswahili, or Luo during first or second visit for antenatal care at a study site.

#### Exclusion criteria

- Indicate they do not intend to receive further antenatal, postnatal, or PMTCT care at the site;
- Are not physically and/or emotionally able to complete the informed consent process to initiate/participate in study (mentally ill, drug abuse, etc.).

### Allocation to study arms

**Map 1:**
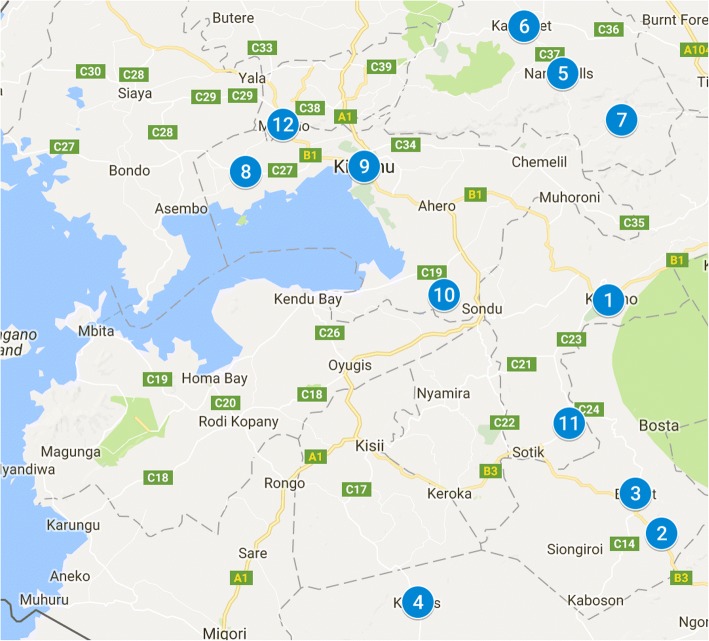
Location of study sites (numbers 1 - 12)

The 12 study sites were formed into six pairs, matched on observed characteristics including geographic location and number of HIV-infected pregnant women presenting for antenatal care (during 2016). Map 1 identifies the basic location for each study site, numbered from 1 to 12, which represents the order sites began enrolment (1 was first, 12 was last). The study site names by study arm are: (SOC Arm/EMMA Arm): 6 = Kapsabet District Hospital/1 = Kericho County Referral Hospital; 11 = Kapkatet District Hospital/4 = Transmara District Hospital; 8 = Kombewa County Hospital/5 = Nandi Hills District Hospital; 12 = Ratta Health Centre/3 = Bomet Health Centre; 2 = Longisa District Hospital/9 = Munyuanda Health Centre; 7 = Meteitei-Sub-district Hospital/10 = Bodi Health Centre. The largest sites were matched together and the study team tried to maintain regional balance between the six counties in which the study is being conducted.

Clinics in the intervention arm provide EMMA services to all patients at the study site, so providers are not blind to their treatment assignment. The study, however, enrolls a subset as study participants for prospective records review. Thus, patients are not blinded but all patients at the intervention sites are provided with EMMA services. After enrollment in the study, individuals receive the same services as provided to all patients at their clinic.

### Participant timeline

**Fig. 1:**
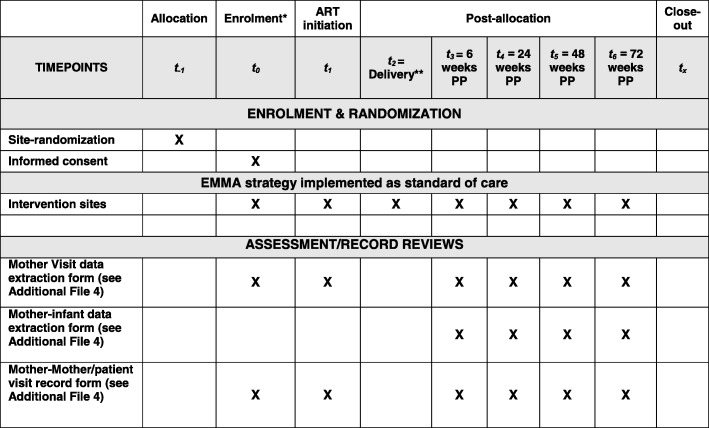
SPIRIT figure (Study timeline)

**Fig. 2:**
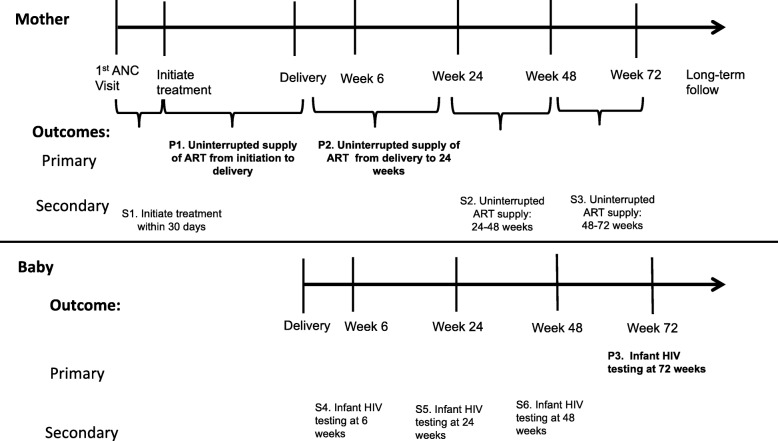
Timeline showing primary (P) and secondary (S) outcomes to be evaluated

The participant timeline is detailed in Fig. 1 (Spirit Figure), with additional detail provided in Fig. 2 discussed below in relation to study outcomes.

#### Recruitment and enrollment

All women with HIV will be informed about the study by the site’s PMTCT clinical staff (nurses and/or Clinical Officers, depending on site) during their first visit for antenatal care. These clinical staff will use study-developed briefing slides to guide this discussion (see Additional file 3 – Briefing Slides). Those interested in participating, where “participation” means the patient allows the study to review their medical record information collected routinely by the site for patient care, will be referred to the study staff at the site. Study staff will then explain the nature of the study, confirm study eligibility, review the informed consent documents, and obtain written informed consent. The study staff member completing consenting procedures will not be the Mentor Mother at the intervention sites. Patients who do not consent to be in the study will be managed as usual at the study site. Those who consent to be in the study will be referred to one of the clinic’s Mentor Mothers once the consenting process is complete. The study will continue enrolling patients until the target sample size for each site is reached.

Mentor Mothers are already part of the PEPFAR supported staff at the study sites. Their recruitment criteria and general assignments for clinics are clearly stipulated in the KMMP NASCOP implementation guidelines [4]. To implement the EMMA strategy, all Mentor Mothers at the intervention study sites will receive EMMA-specific training as well as refresher training on adherence counseling and psychosocial support (based on the original Mentor Mother curriculum). In addition to EMMA-specific training, since this is a research study, all staff participating in the study will undergo ethics training and study specific research training (included CITI certification).

#### Follow-up

After enrollment in the study, other than interactions with the Mentor Mother as part of EMMA activities in the intervention arm, study staff will have no further contact with study participants. Follow-up for data extraction from medical records will continue through 18 months post-partum for each mother enrolled (and 18 months of age for her child).

### Outcomes measures

#### Primary outcomes

Study outcomes are organized along the PMTCT cascade of care (see Fig. 2), from the first ANC visit through 72 weeks post-partum to evaluate adherence to clinic visits (to receive continuous supplies of ART and for infant HIV testing) and retention in care. Successful implementation of the PMTCT services implies that: (1) pregnant women with HIV initiate life-long ART with minimal delay after their first ANC visit (and women already on ART should continue on ART); (2) they continue on treatment through delivery; (3) they continue on treatment after delivery and the cessation of breastfeeding (and continuing for life for their health); and (4) their child completes HIV testing at 6, 24, 48, and 72 weeks so that the infant is known to be uninfected after the cessation of breast feeding or that the infant is diagnosed and initiated on ART early should he/she become HIV-positive.

The three primary outcomes are:

- P1: the proportion of HIV-positive pregnant women who receive an uninterrupted supply of ART from treatment initiation to delivery;
- P2: the proportion of HIV-positive pregnant women who receive an uninterrupted supply of ART from delivery to 24 weeks post-partum;
- P3: the proportion of babies with known HIV results by 72 (± 4) weeks

To operationalize “uninterrupted supply” for primary outcomes 1 and 2, we will follow Haas et al. [15] and calculate the percentage of days covered by drugs dispensed, with ≥ 90% of days covered deemed as uninterrupted.

#### Secondary outcomes

- S1: the proportion of women initiating ART within 30 days of their first ANC visit;
- S2: the proportion of pregnant women with HIV receiving an uninterrupted supply of ART at 24–48 weeks post-partum;
- S3: the proportion of pregnant women with HIV receiving an uninterrupted supply of ART at 48–72 weeks post-partum;
- S4: the proportion of infants completing HIV testing at 6 weeks (± 4 weeks), along with the proportion testing positive for HIV;
- S5: the proportion of infants who complete HIV testing at 24 weeks (± 4 weeks), along with the proportion testing positive for HIV;
- S6: the proportion completing HIV testing at 48 weeks (± 4 weeks), along with the proportion testing positive for HIV.

### Sample size

Each primary outcome is a proportion and the sample size (360 total, 180 per arm) was developed to be adequate to detect at least a 25 percentage-point improvement in each primary outcome between the two study groups (Arms SOC and SOC + EMMA). To begin, ignoring possible cluster effects, Table 1 first shows the sample size needed to detect a 0.25 proportional improvement between the two study groups, when the base proportion is in the range of 0.30–0.40 (with 5% significance, 80% power, assuming a two-sided test, using sampsi command in Stata 11). Baseline proportions > 0.4 have smaller estimated sample sizes than for 0.4, so they are not reported. Table 1 also shows how the sample size increases when possible clustering effects are included into the sample size calculation (using sampclus in Stata 11). With 12 possible clusters, and an intra-cluster correlation coefficient of 0.04 and 0.05, the sample size increases by 76–130% with six sites allocated to each study arm (and 20–27 patients per cluster).

**Table 1:** Sample sizes to detect 25 percentage point difference in study arms (5% significance, 80% power)

SOC	SOC + EMMA	Random sample size SOC/SOC + EMA	Clusters (n)	Intra-cluster correlation (rho)	Total sample size	Per cluster	Implied design effect
0.30	0.55	68/68	12	0.04	120/120	20	1.76
0.40	0.65	70/70	12	0.04	126/126	21	1.80
0.40	0.65	70/70	12	0.05	161/161	27	2.30

Information does not exist at the sites to estimate the needed baseline proportion (e.g. for Arm SOC). In addition, data on patient progression through the PMTCT cascade are notoriously lacking. For example, PEPFAR indicators show the number of infants (< 2 months of age or aged 2–12 months) receiving an HIV test in a calendar month, but the denominator, which would be the number of mothers with HIV who came to the site for a first ANC visit in the past and enough time has elapsed so that they are at least six weeks post-partum, is not reported. Based on the information in Table 1, we propose a sample size of approximately 30 patients per cluster. This number, which is about 10% larger than largest sample size number in Table 1, accounts for patient attrition from the study that is not addressed as study. For example, some patients enrolled in the study may transfer to another site at some point during the study. Adverse pregnancy outcomes unrelated to the study are also possible (e.g. miscarriage), for which infant outcomes would not exist and the women would be transferred from the site’s MCH clinic to the general HIV clinic for further HIV-related care.

### Data collection methods

Follow-up for data extraction will be passive, by medical record review only, and continue through 72 weeks postpartum. This period of time is needed to evaluate long-term retention in care through cessation of breastfeeding, which includes the 72-week (18-month) postnatal period when the patient is managed at the MCH clinic for ART services.

All information required for the evaluation will be extracted from information on patient care routinely collected by the sites in both study arms as well as the additional information routinely collected by the Mentor Mother providing EMMA as part of her interactions with patients.

Before the study begins, study staff will review each site’s patient record system and related data collection procedures and address any systematic problems that will hinder study data collection (e.g. failure to enter visit dates, drugs prescribed, recording negative HIV test results rather than leaving information blank, which have been common problems we have encountered during prior studies at these and other sites).

The study will use three clinical records forms (CRF) for extracting information from the patient’s medical file through 72 weeks post-partum (up to 76 weeks to confirm final HIV test completion for the infant). The three CRFs are included in Additional file 4 – EMMA CRFs.

- The EMMA study mother’s visit data extraction form (see Additional file 4). This form contains the basic information to be extracted from the individual’s medical record for visits to the clinic. This process allows us to create a longitudinal dataset (multiple visits for each patient) to complete the data analysis to create the primary and secondary study outcomes for each study participant (the mother);
- The EMMA study mother-infant data extraction form (see Additional file 4). This form contains the basic information needed to assess primary and secondary outcomes for the infant (date of delivery, dates of HIV tests, etc.). Date of delivery from the mother-infant form is also then merged into the mother’s longitudinal database to create the key primary and secondary outcomes for the mother;
- The EMMA study Mentor Mother/patient visit record form (see Additional file 4). At the sites providing the EMMA intervention, Mentor Mothers will complete the Mentor Mother/patient visit record form after each exit visit with a mother. This form will just record the date of the visit, the mother’s name and clinic ID number, a field to indicate if an SMS message was scheduled to be sent to the mother at this visit, and a field to indicate if a reminder was set up on the mother’s phone. The Mentor Mother will complete this form for all patients at the site during the study intervention period. These forms then become part of the patient’s medical file. The study will extract data from this form for patients enrolled in the study.

### Data entry and storage

Each subject will be assigned a four-digit study identification number to be used to store and analyze data. The first two digits will indicate the study site (01–12) and the next two digits will be assigned sequentially as patients enroll in the study (01–30). KEMRI/WRP data entry clerks will input the relevant information from medical records (as outlined above) to the patient visit database (the study database will be developed using ClinPlus by the Data Coordinating and Analysis Center of the US Military HIV Research Program (MHRP)).

Password-protected computers for data entry are in lockable offices at the KEMRI/WRP headquarters in Kericho and the rooms are locked when not in use. All forms used for extracting information from clinical files will be stored in locked cabinets within locked offices.

The study database will be managed and maintained on a MHRP secure server by the Data Computing and Analysis Center (DCAC) in Bethesda, MD. The study database will be password-protected, with access limited to the study team. The linking file, which will contain name, clinic ID, and study ID, will be stored separately. We will run data cleaning routines regularly and generate queries to return to the site for resolution as needed.

### Data analysis

All analyses will be by intention to treat: patients will be analyzed according to the intervention arm for their site. At the individual level, the primary outcomes are dichotomous variables (e.g. 1 if the women received an uninterrupted supply of ART from initiation through delivery, 0 if not). For each study arm, these dichotomous outcomes can be summarized as proportions (the proportion pregnant women with HIV who receive an uninterrupted supply of ART from treatment initiation to delivery).

The analysis will begin with a simple comparison of the proportion of successes for each outcome (e.g. received an uninterrupted supply of ART from initiating to delivery) by study arm and crude risk ratios and 95% confidence intervals stratified by study arm will be reported. To estimate the risk difference between study arms, a linear probability model will be used with t-statistics and *p* values adjusted for the small number of clusters (study clinics). A linear probability model is appropriate for estimating impacts when treatment status is a binary (as in this study). [16] For inference, the *clustse* analysis in STATA will be used to adjust t-statistics and *p* values for the small number of clusters. *Clustse* implements the cluster-adjusted t-statistics described in [17] and wild cluster bootstrap procedure described in [18].

Because we will not be randomizing large numbers of patients during the intervention period, it is possible that some covariates may be imbalanced by study arm. To adjust for potential differences in such covariates, multivariate linear probability models will be used to estimate adjusted risk differences. Adjusted analyses will include covariates that are unevenly distributed across study groups for each outcome and could plausibly affect study outcomes. These include patient characteristics (e.g. age, weeks of gestation at first ANC visit, parity) and site characteristics. Statistical analyses will be completed using STATA. We will use similar analytic methods for analyzing secondary outcomes.

In addition, there is debate in the literature as to how the pair-matched design should be accounted for in the analysis to avoid bias. [19–22] To this end, supportive analyses will use alternative strategies to account for the pair-matched design in order to evaluate robustness of model-based estimators that do not explicitly account for the pair-matched design.

For the analysis of costs of implementing the EMMA intervention, we will use standard program costing analysis methods to assess the additional costs to the health facilities of providing the EMMA intervention [23–25]. Only program records are required for the cost analysis (e.g. training costs for Mentor Mothers, costs for phones and airtime, etc.). No human subject data are required for the evaluation of costs. See Additional file 5 for an overview of the main steps to be followed for completing the cost analysis.

### Study monitoring

All of the patient-level data used for this study will be extracted from information routinely collected at the study sites and contained in patient medical records (paper files for the mother’s visit extraction form and the mother-infant form; electronic file for the Mentor Mother/Patient Encounter).

As these forms are completed over time based on the study schedule, they will be checked for completeness by the study coordinator. If any changes to forms are made, the original data will be left and crossed out and the corrected data will be written next to it with a signature and date of the person who made the change. The Study Coordinator will provide monthly updates on study enrollment numbers to the PI for monitoring enrollment progress.

Given the largely observational nature of this study, monitoring will focus upon, but not be limited to, informed consent, patient confidentiality, regulatory components (e.g. IRB approvals), and IRB reports. The PI will conduct monitoring activities every six months, in which an experienced study coordinator and data manager working with Walter Reed/KEMRI program in Kericho will review 100% of informed consents and a 10% sample of all newly completed forms and entered data since the last monitoring visit and compare to original medical records. If any issues are found during regulatory monitoring activities, a one-time monitoring visit will be done within a period of three months and staff will be retrained. For this minimal risk study addressing behavioral and staff management issues, a data monitoring committee was not organized [26].

## Discussion

The purpose of the EMMA intervention is to maximize retention in care and adherence to scheduled visits for pregnant woman with HIV and their infants, from the mother’s first ANC visit through 18 months post-partum. The EMMA intervention is designed to support implementation of existing Kenyan policies for PMTCT service delivery, including the Mentor Mother program. The basic strategy outlined above (EMMA) will be implemented at the site level and all adult pregnant women with HIV at the study site will be offered Mentor Mother services throughout the duration of the study (the existing Mentor Mothers services at the comparison sites and the enhanced Mentor Mother services [EMMA] at the intervention sites). A Mentor Mother patient interaction guide will be developed in collaboration with PMTCT staff to assist with providing structure to the exit counseling session. In effect, EMMA provides specific structure to the “One to One Education and Mentorship” envisioned in the Kenya Mentor Mother Program [4]. Importantly, a low-cost intervention, EMMA adds to existing PMTCT operational costs to purchase airtime for sending messages. Yet, if successful, the EMMA strategy has the potential to improve PMTCT service delivery with minor additional costs beyond the basic mother-mentor program and support global goals to eliminate mother to child transmission.

In discussions with the site and PEPFAR program staff, the current intervention and evaluation design as proposed are the most logical to consider and logistically feasible. Because of the importance of site randomization for evaluating the impacts of an intervention in this setting, it is not feasible to have a three-armed strategy trial within the study funding period, where: (1) certain sites receive no intervention; (2) some receive EMMA but without text messaging/reminders; and (3) others receive EMMA. The EMMA trial findings will not be generalizable to woman-child dyads served by clinics without integrated ART-MCH services.

We expect intervention and research activities to be implemented within the timeline set by the study team in discussions with the site and PEPFAR program staff to accommodate expected activity duration and any extraneous circumstances (e.g. the presidential election, post-election unrest), potential to introduce disruptions (in routine clinical operations) and delays.

### Dissemination

We plan to disseminate EMMA trial findings via peer-reviewed academic journals and conferences. We will make available for public use the trial protocol and de-identified data after publishing results for the primary and secondary outcomes. Results will also be provided to and discussed with study sites, the SRVP, and the Kenyan MOH. For manuscripts, the criteria for authorship will be based on standard journal requirements. Professional writers will not be used.

### Trial status

The current approved version of the protocol is Version 5.7, 6 March 2017.

Recruitment began on 17 March 2017. Enrollment was then put on hold during April–July 2017 pending PEPFAR assessment if research studies would continue to be funded.

Recruitment is expected to be complete in February 2018.

## Additional files

Additional file 1: SPIRIT Checklist. Checklist of the Standard Protocol Items: Recommendations for Interventional Trials (SPIRIT) guidelines. (DOCX 66 kb)

Additional file 2: Mentor Mother Guide. (DOC 42 kb)

Additional file 3: Briefing Slides. (PPTX 219 kb)

Additional file 4: EMMA CRFs (Mother visit data extraction form; mother-infant data extraction form; Mentor-Mother/patient visit record form). (PDF 176 kb)

Additional file 5: Overview of costing analysis. (PPTX 94 kb)

## Abbreviations

ANC: Antenatal care
ART: Antiretroviral therapy
BU: Boston University
CCR: Center for Clinical Research
CITI: Collaborative Institutional Training Initiative
DCAC: Data Coordinating and Analysis Centre
DoD: Department of Defense
EMMA: Enhanced Mentor Mother ProgrAm
HIV: Human immunodeficiency virus
ICF: Informed consent forms
IEC: Independent Ethics Committees
IRB: Institutional Review Boards
KEMRI: Kenya Medical Research Institute
KMMP: Kenya Mentor Mother Program
MCH: Maternal and child health
MHRP: US Military HIV Research Program
M-PESA: Mobile money transfer service (not an abbreviation)
PEPFAR: President’s Emergency Plan For AIDS Relief
PMTCT: Prevention of mother-to-child transmission
SC: Scientific Committee
SERU: Scientific and Ethics Review Unit
SOC: Standard of care
SRVP: South Rift Valley Program
USAMRMC: United States Army Medical Research and Material Command
WRAIR: Walter Reed Army Institute of Research
WRP: Walter Reed Program
