# Supplementary material for: The Enhanced Mentor Mother ProgrAm (EMMA) for the prevention of mother-to-child transmission of HIV in Kenya: study protocol for a cluster randomized controlled trial
Source: Trials. 2018 Oct 30;19:594. doi: 10.1186/s13063-018-2975-y (PMC6208066; doi:10.1186/s13063-018-2975-y)
Supplement: Supplementary file 2 — Mentor Mother Guide. (DOC 42 kb) [file 13063_2018_2975_MOESM2_ESM.doc]

**EMMA Mentor Mother/ Patient Interaction Guide.**

**Selection of Mentor Mother:**

1. Women living with HIV
2. Should have gone through PMTCT program at some point during a previous pregnancy

**Mother Specific** (provide one-on-one peer education and psychosocial support to mother and partner)

1. Are you attending the clinic for the first time? (Yes/ No)
2. Did you receive ARVs during this visit? (Yes/ No)
3. If yes, check adherence between visits.
4. Tell me how you plan to take your ARVs as per the discussion in the clinic today.
5. Reinforce adherence counseling based on no.3 & 4.
6. Did you discuss the importance of taking ARVs with your Doctor? (Yes/ No)
7. From the discussion with your Doctor today, why is it important to come to the clinic as scheduled?
8. Reinforce HIV education as need be based on No.6.
9. Who have you disclosed your status to (Partner\Child\ Other\ Not yet)?
10. Encourage partner involvement as appropriate.
11. Male partner HIV status (this pregnancy)- Positive \Negative\Unknown\No partner
12. Planning to deliver in Facility (Yes/No) – if ANC visit.
13. Encourage to deliver in health facility (ANC).
14. Discuss Family planning and encourage use of modern methods
15. Discuss Feeding options and if further knowledge is required.
16. HIV basic education (educational support) and nutrition education as need be (knowledge of adequate maternal nutrition)
17. Discuss if patient has Stigma and discrimination issues. (Motivate behavior change and help mother deal with any existing stigma that might hinder their follow-up.)
18. Give psychosocial support. Share personal experiences as appropriate.
19. When is your next clinic appointment

**Infant**

1. Is the child attending clinic for the first time since birth? (Yes/ No)
2. Was your child tested for HIV during this visit during this visit? (Yes/ No)
3. Is your child on septrin? (Yes/ No)
4. Is your child on ARVS? (Yes/ No)
5. Assess baby adherence and any issues mother has (discuss.)
6. Discuss child health and immunizations (check schedule follow-up)

**Adherence to patient follow-up:**

1. Explain to the mother that EMMA staff would like to help them keep their schedule and follow-up at the clinic for sake of both mother and babies help. Explain the tool used to remind the mother and discuss follow-up options.
2. Schedule a reminded sms and other tracking for the patient. If patient agrees set a reminder on their phones esp. for patients who do not want telephonic follow-up.
3. Ensure the contact tracing form is updated periodically to ensure contact information is up to date.
