# Supplementary material for: The Enhanced Mentor Mother ProgrAm (EMMA) for the prevention of mother-to-child transmission of HIV in Kenya: study protocol for a cluster randomized controlled trial
Source: Trials. 2018 Oct 30;19:594. doi: 10.1186/s13063-018-2975-y (PMC6208066; doi:10.1186/s13063-018-2975-y)
Supplement: Supplementary file 3 — Briefing Slides. (PPTX 219 kb) [file 13063_2018_2975_MOESM3_ESM.pptx]

## Slide 1
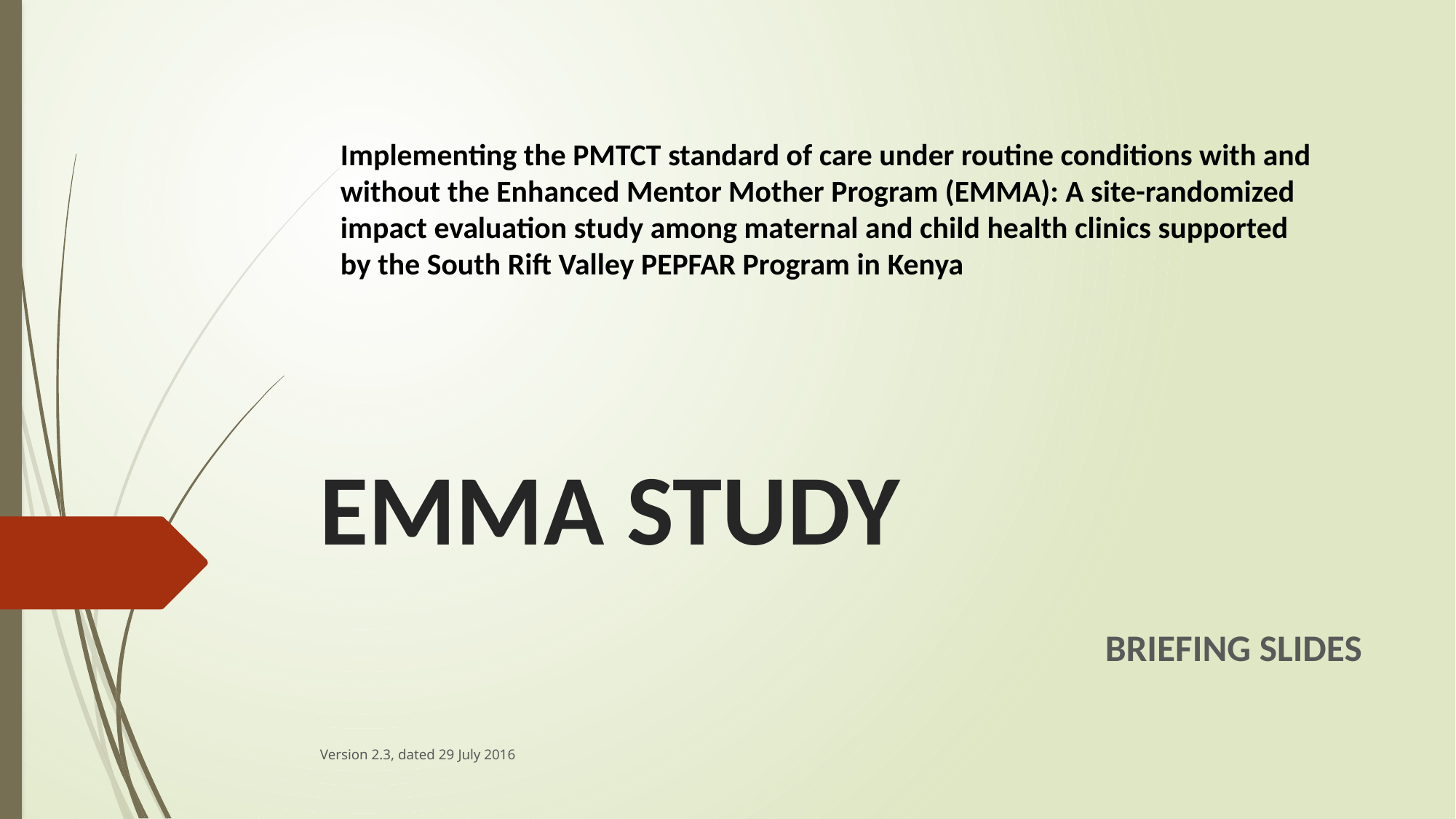

Implementing the PMTCT standard of care under routine conditions with and without the Enhanced Mentor Mother Program (EMMA): A site-randomized impact evaluation study among maternal and child health clinics supported by the South Rift Valley PEPFAR Program in Kenya
# EMMA STUDY
BRIEFING SLIDES
Version 2.3, dated 29 July 2016

## Slide 2
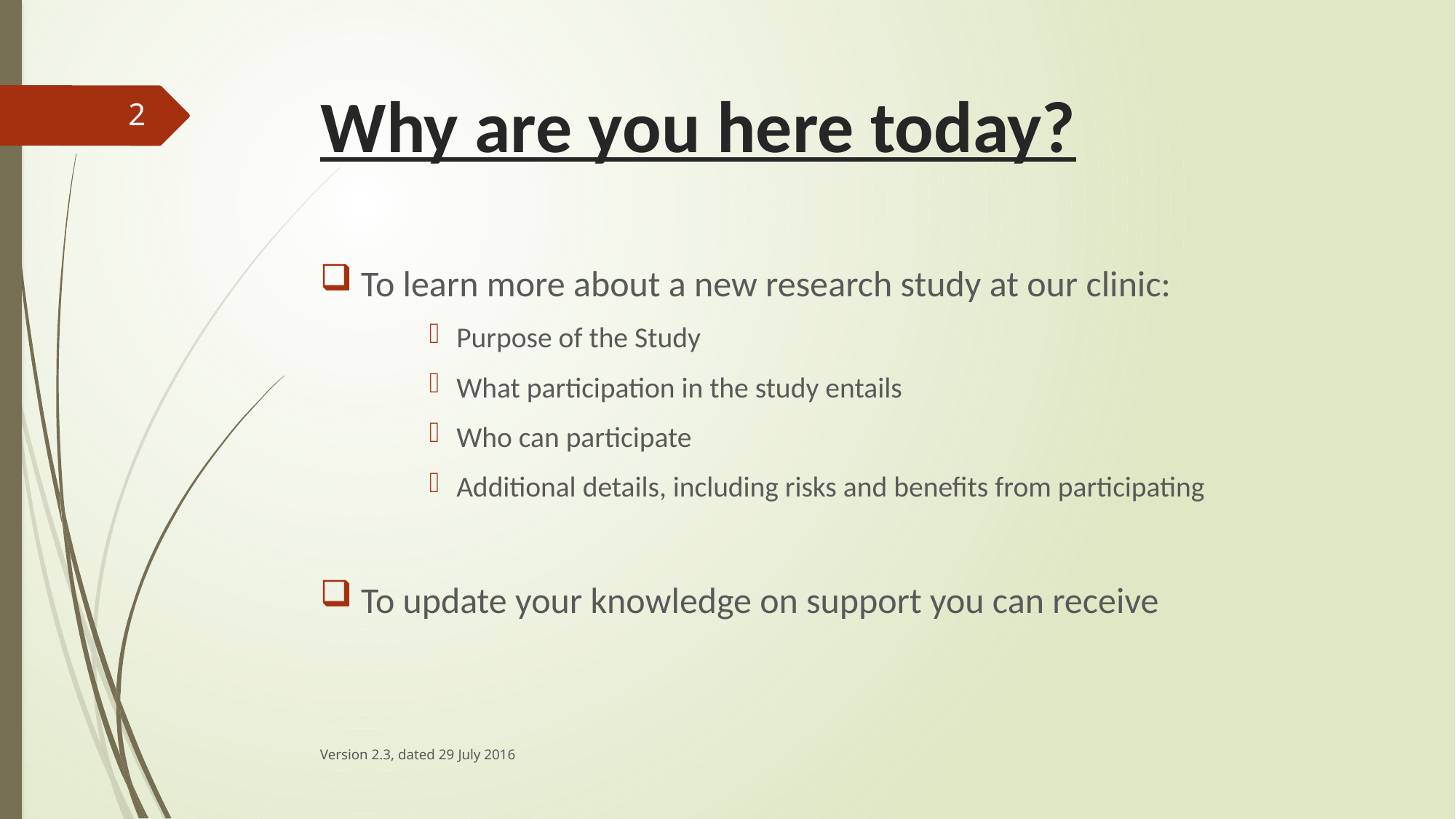

# Why are you here today?
2
To learn more about a new research study at our clinic:
Purpose of the Study
What participation in the study entails
Who can participate
Additional details, including risks and benefits from participating
To update your knowledge on support you can receive
Version 2.3, dated 29 July 2016

## Slide 3
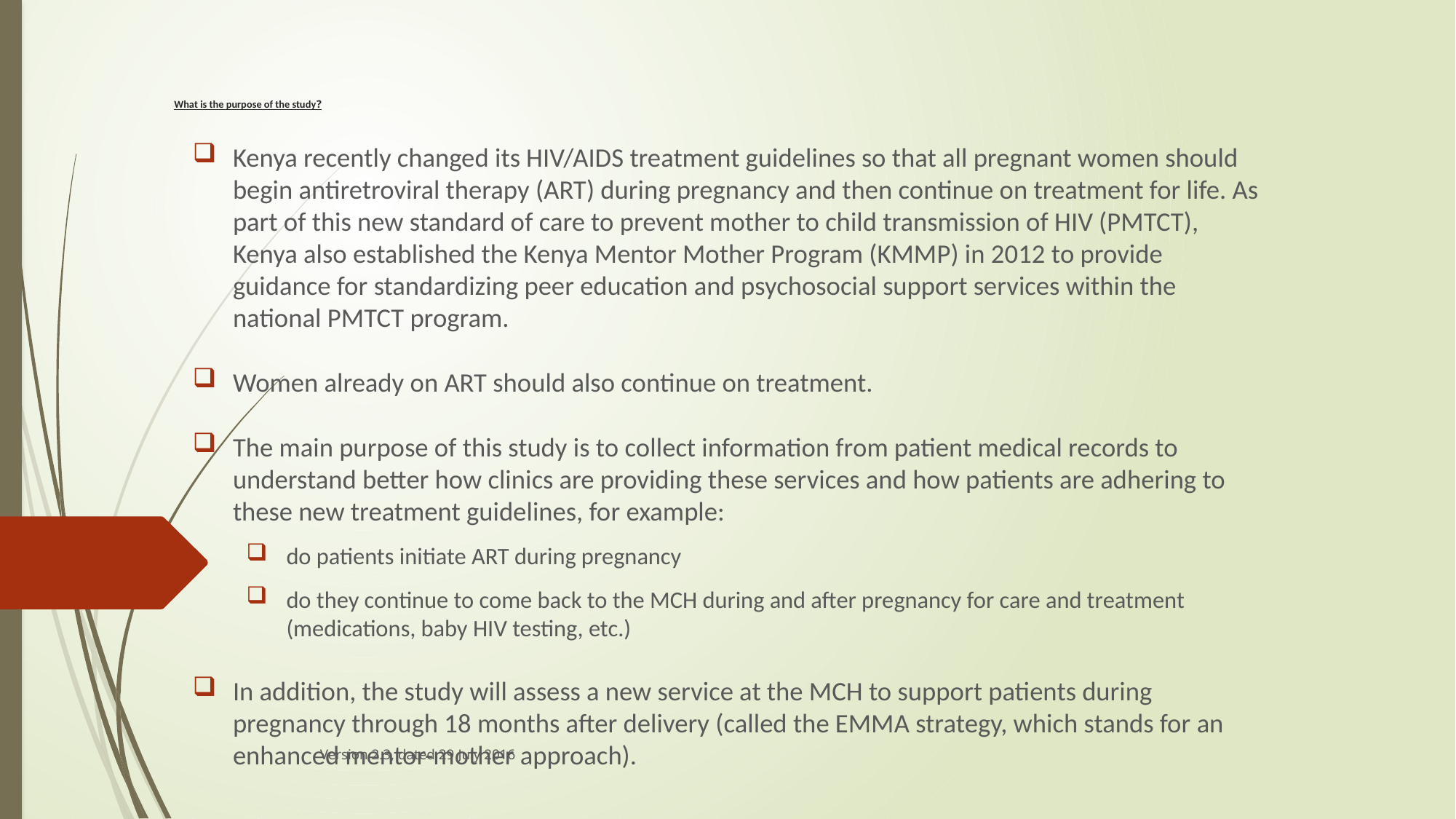

# What is the purpose of the study?
Kenya recently changed its HIV/AIDS treatment guidelines so that all pregnant women should begin antiretroviral therapy (ART) during pregnancy and then continue on treatment for life. As part of this new standard of care to prevent mother to child transmission of HIV (PMTCT), Kenya also established the Kenya Mentor Mother Program (KMMP) in 2012 to provide guidance for standardizing peer education and psychosocial support services within the national PMTCT program.
Women already on ART should also continue on treatment.
The main purpose of this study is to collect information from patient medical records to understand better how clinics are providing these services and how patients are adhering to these new treatment guidelines, for example:
do patients initiate ART during pregnancy
do they continue to come back to the MCH during and after pregnancy for care and treatment (medications, baby HIV testing, etc.)
In addition, the study will assess a new service at the MCH to support patients during pregnancy through 18 months after delivery (called the EMMA strategy, which stands for an enhanced mentor-mother approach).
Version 2.3, dated 29 July 2016

## Slide 4
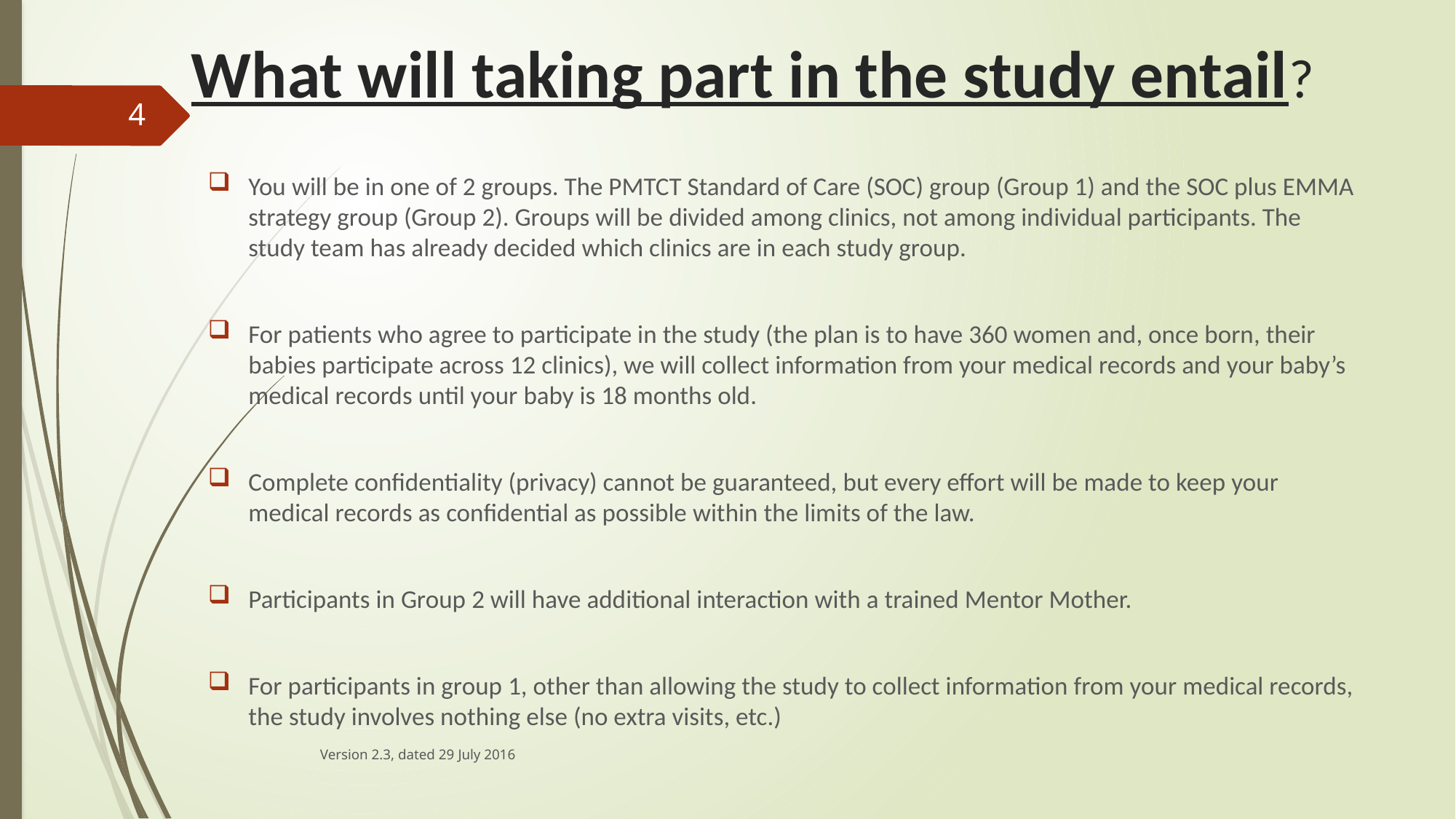

# What will taking part in the study entail?
4
You will be in one of 2 groups. The PMTCT Standard of Care (SOC) group (Group 1) and the SOC plus EMMA strategy group (Group 2). Groups will be divided among clinics, not among individual participants. The study team has already decided which clinics are in each study group.
For patients who agree to participate in the study (the plan is to have 360 women and, once born, their babies participate across 12 clinics), we will collect information from your medical records and your baby’s medical records until your baby is 18 months old.
Complete confidentiality (privacy) cannot be guaranteed, but every effort will be made to keep your medical records as confidential as possible within the limits of the law.
Participants in Group 2 will have additional interaction with a trained Mentor Mother.
For participants in group 1, other than allowing the study to collect information from your medical records, the study involves nothing else (no extra visits, etc.)
Version 2.3, dated 29 July 2016

## Slide 5
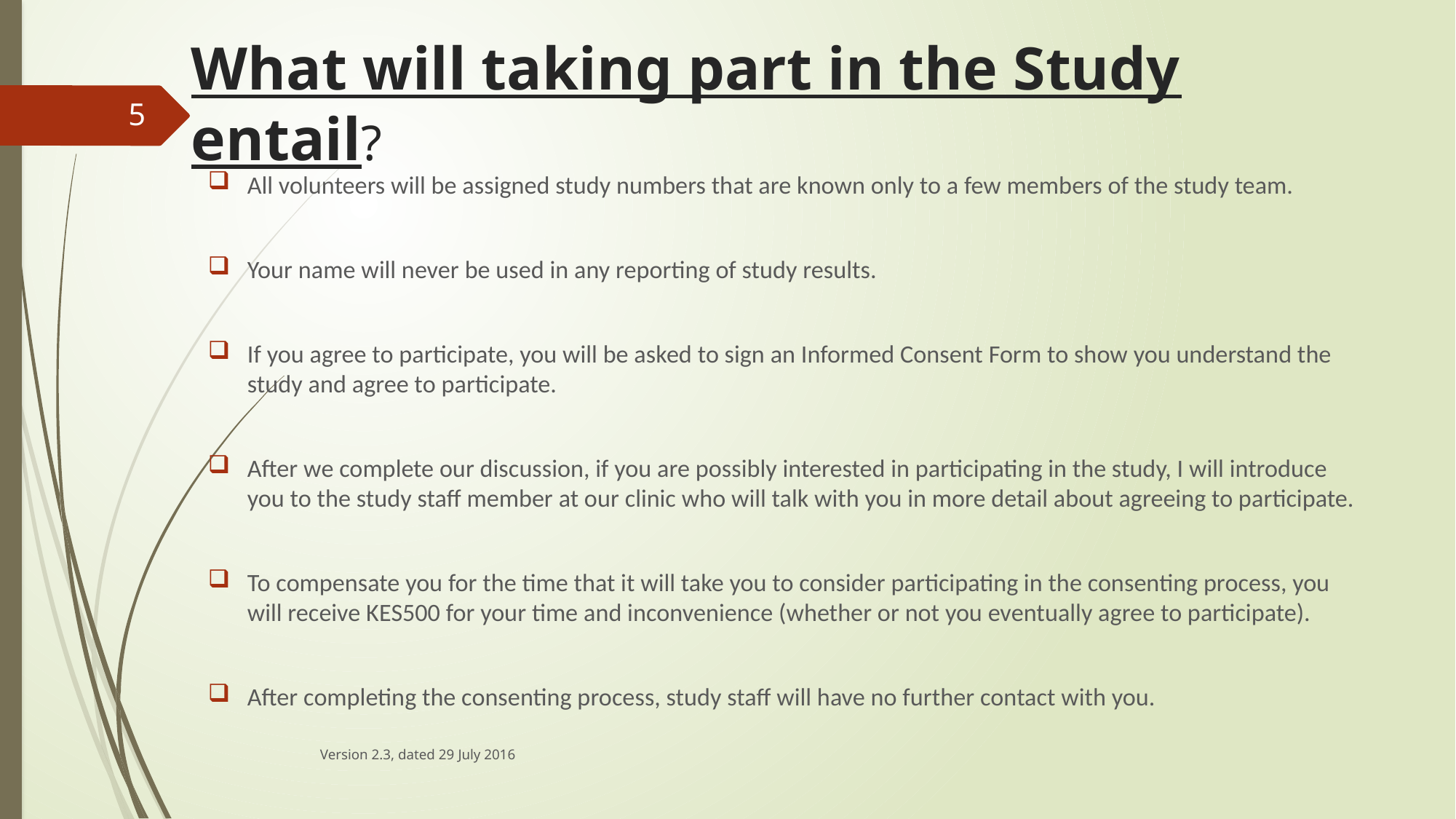

# What will taking part in the Study entail?
5
All volunteers will be assigned study numbers that are known only to a few members of the study team.
Your name will never be used in any reporting of study results.
If you agree to participate, you will be asked to sign an Informed Consent Form to show you understand the study and agree to participate.
After we complete our discussion, if you are possibly interested in participating in the study, I will introduce you to the study staff member at our clinic who will talk with you in more detail about agreeing to participate.
To compensate you for the time that it will take you to consider participating in the consenting process, you will receive KES500 for your time and inconvenience (whether or not you eventually agree to participate).
After completing the consenting process, study staff will have no further contact with you.
Version 2.3, dated 29 July 2016

## Slide 6
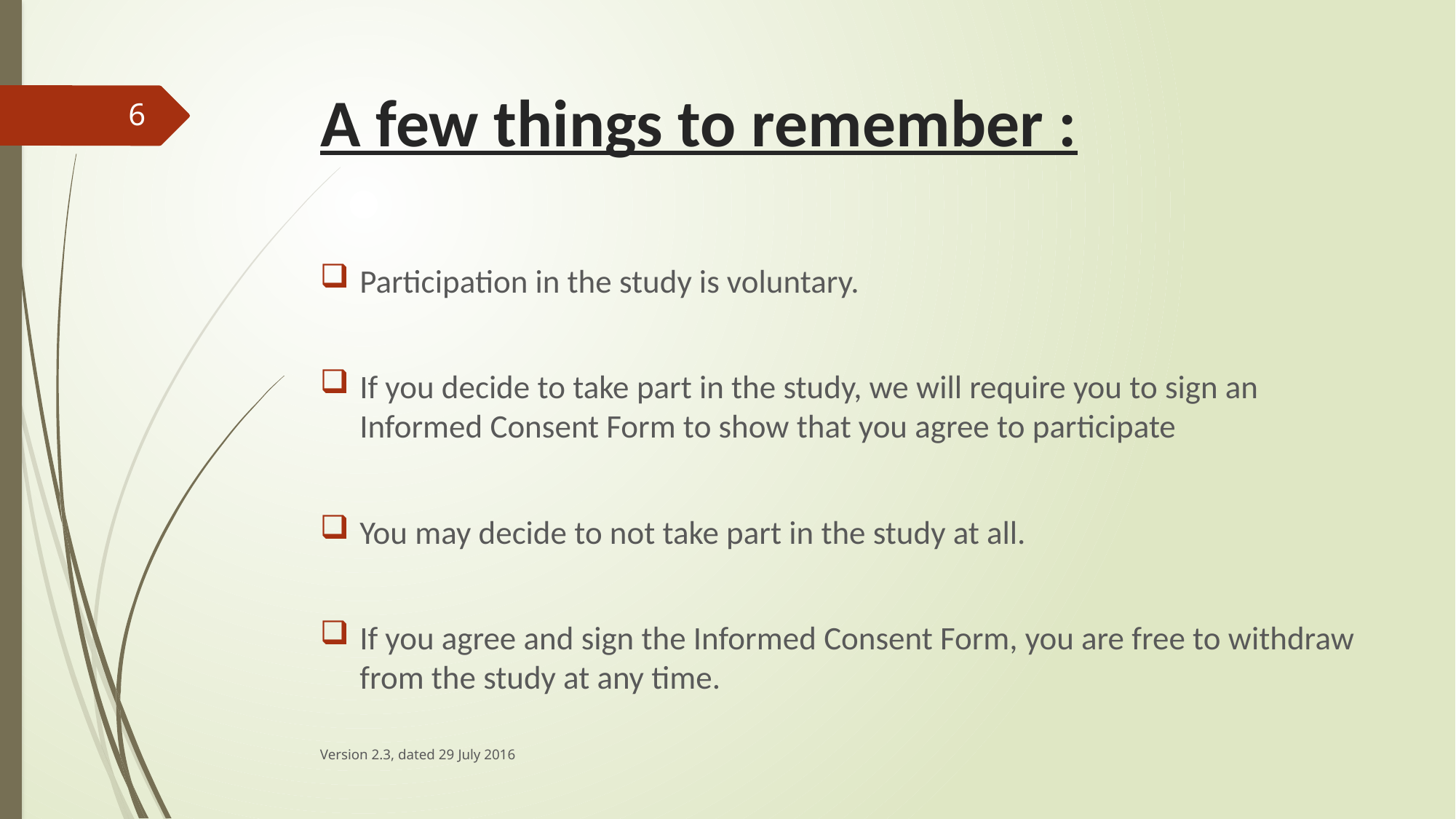

# A few things to remember :
6
Participation in the study is voluntary.
If you decide to take part in the study, we will require you to sign an Informed Consent Form to show that you agree to participate
You may decide to not take part in the study at all.
If you agree and sign the Informed Consent Form, you are free to withdraw from the study at any time.
Version 2.3, dated 29 July 2016

## Slide 7
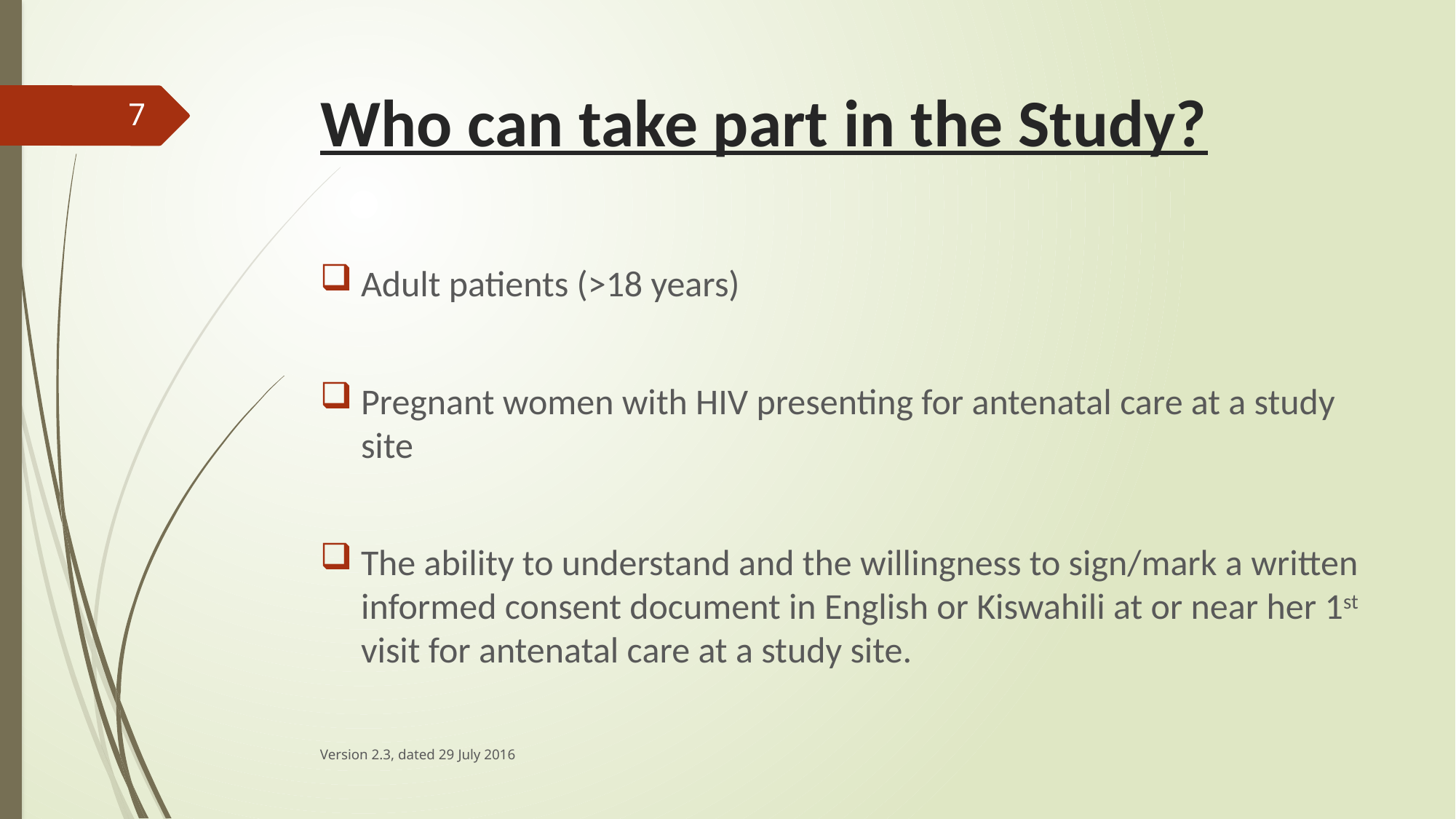

# Who can take part in the Study?
7
Adult patients (>18 years)
Pregnant women with HIV presenting for antenatal care at a study site
The ability to understand and the willingness to sign/mark a written informed consent document in English or Kiswahili at or near her 1st visit for antenatal care at a study site.
Version 2.3, dated 29 July 2016

## Slide 8
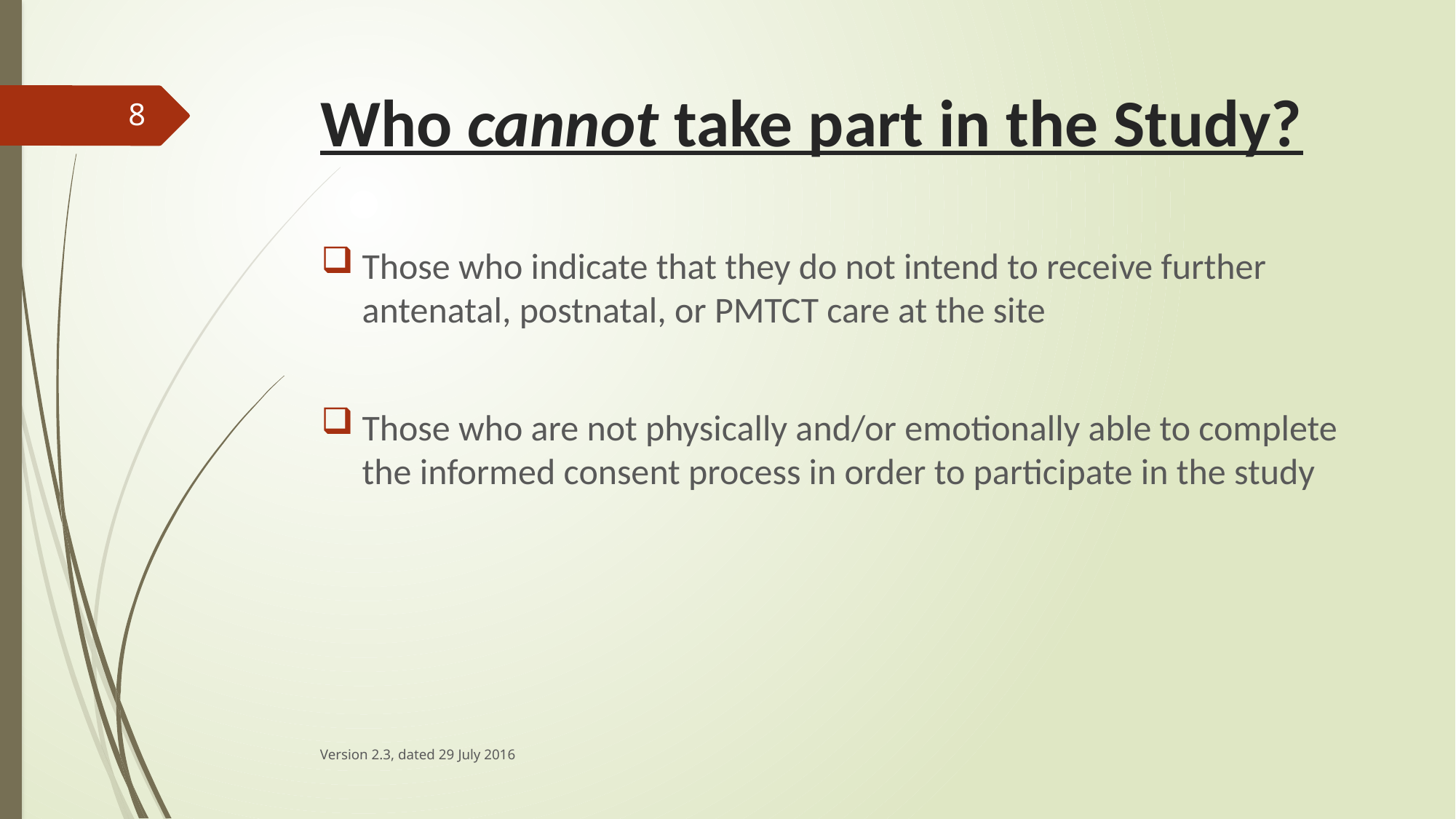

# Who cannot take part in the Study?
8
Those who indicate that they do not intend to receive further antenatal, postnatal, or PMTCT care at the site
Those who are not physically and/or emotionally able to complete the informed consent process in order to participate in the study
Version 2.3, dated 29 July 2016

## Slide 9
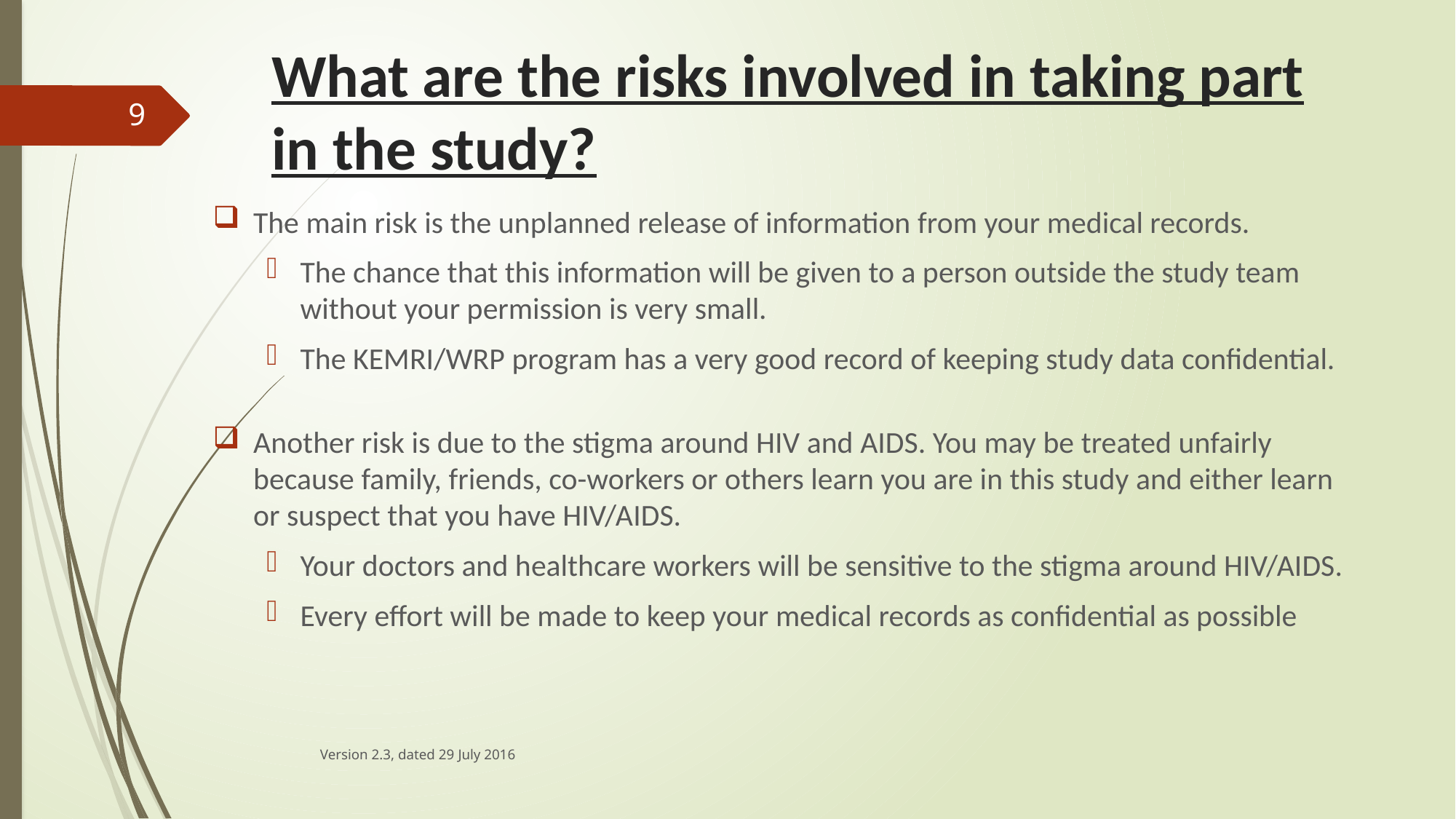

# What are the risks involved in taking part in the study?
9
The main risk is the unplanned release of information from your medical records.
The chance that this information will be given to a person outside the study team without your permission is very small.
The KEMRI/WRP program has a very good record of keeping study data confidential.
Another risk is due to the stigma around HIV and AIDS. You may be treated unfairly because family, friends, co-workers or others learn you are in this study and either learn or suspect that you have HIV/AIDS.
Your doctors and healthcare workers will be sensitive to the stigma around HIV/AIDS.
Every effort will be made to keep your medical records as confidential as possible
Version 2.3, dated 29 July 2016

## Slide 10
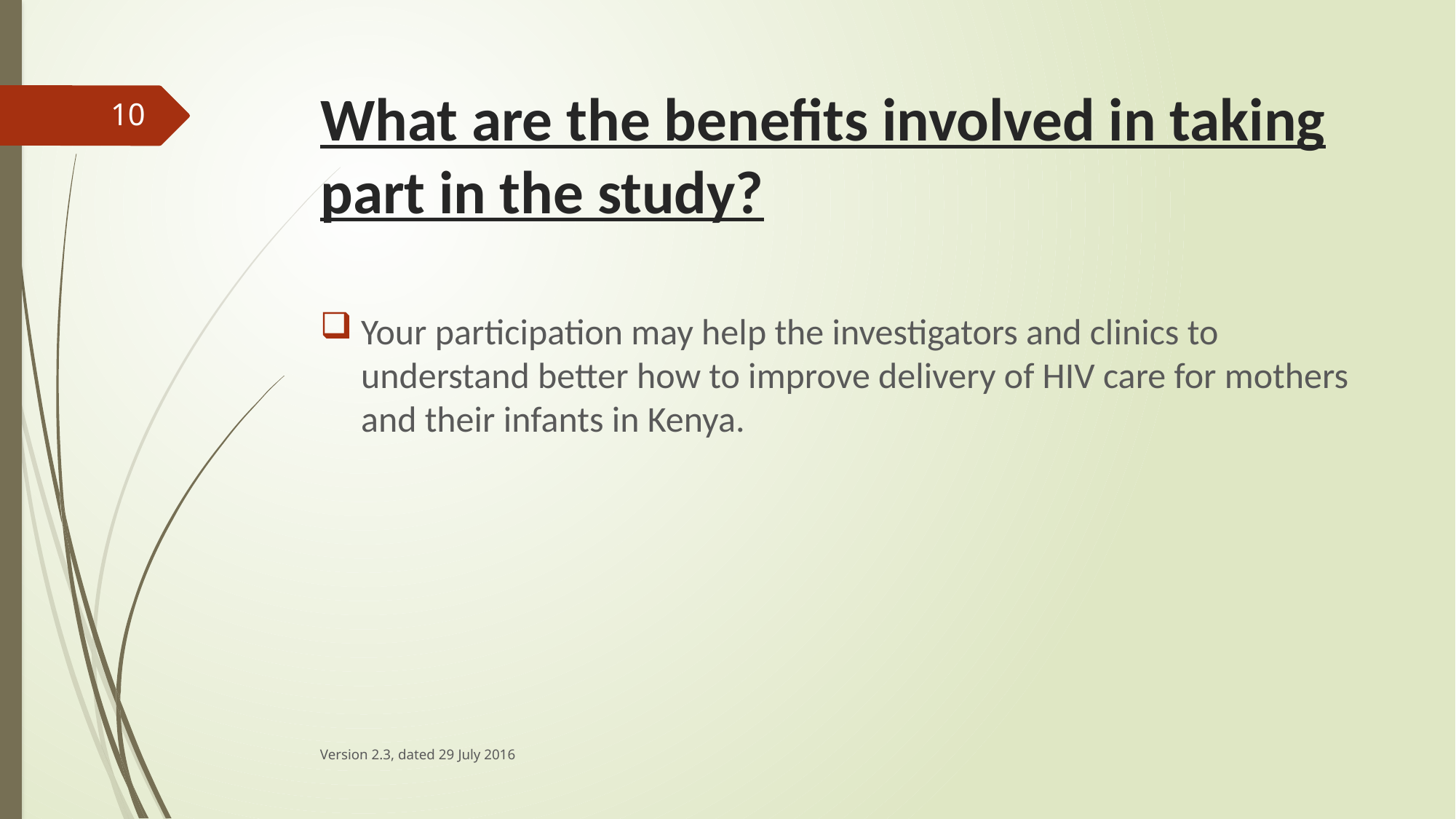

# What are the benefits involved in taking part in the study?
10
Your participation may help the investigators and clinics to understand better how to improve delivery of HIV care for mothers and their infants in Kenya.
Version 2.3, dated 29 July 2016

## Slide 11
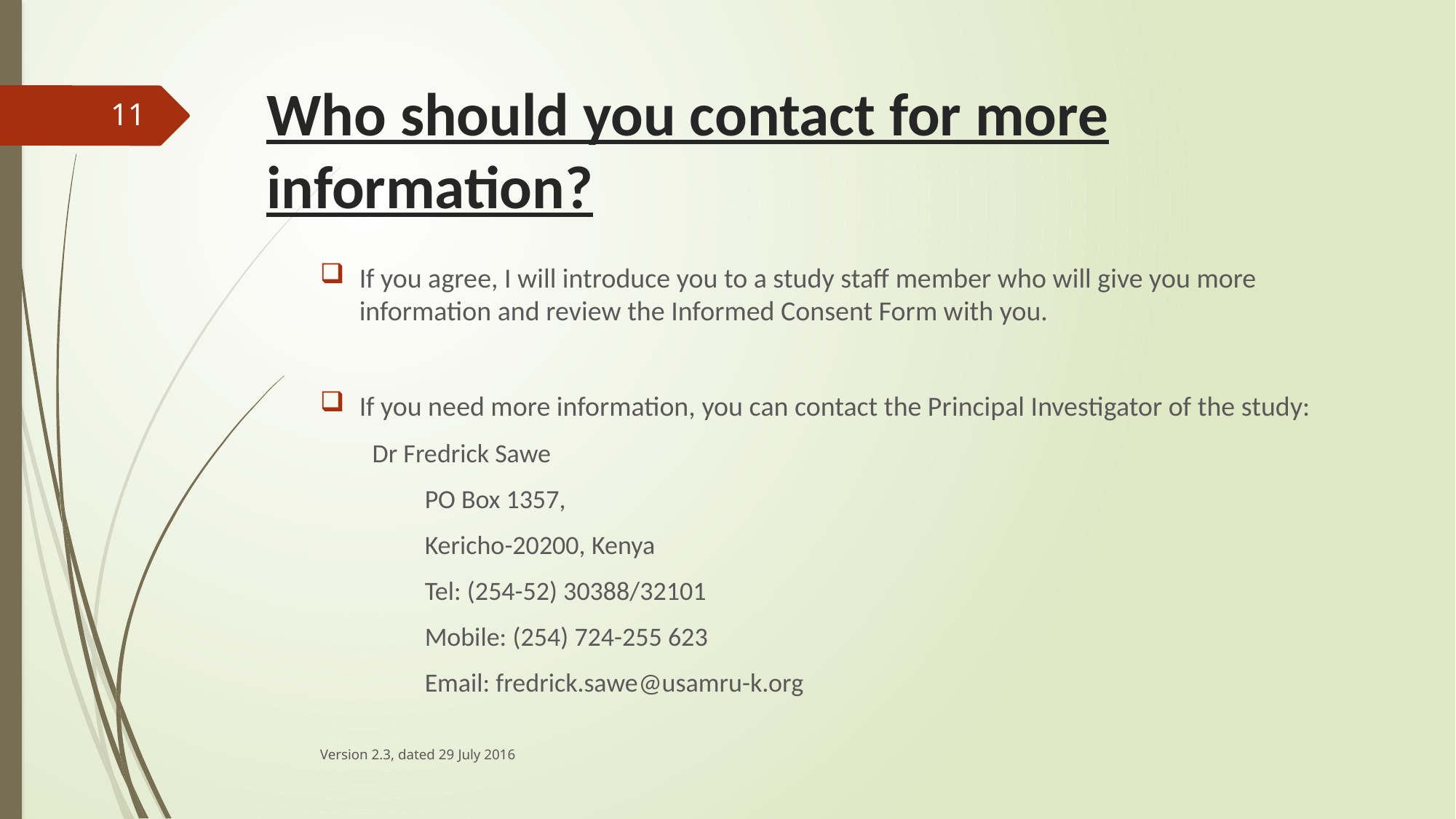

# Who should you contact for more information?
11
If you agree, I will introduce you to a study staff member who will give you more information and review the Informed Consent Form with you.
If you need more information, you can contact the Principal Investigator of the study:
Dr Fredrick Sawe
	PO Box 1357,
	Kericho-20200, Kenya
	Tel: (254-52) 30388/32101
	Mobile: (254) 724-255 623
	Email: fredrick.sawe@usamru-k.org
Version 2.3, dated 29 July 2016

## Slide 12
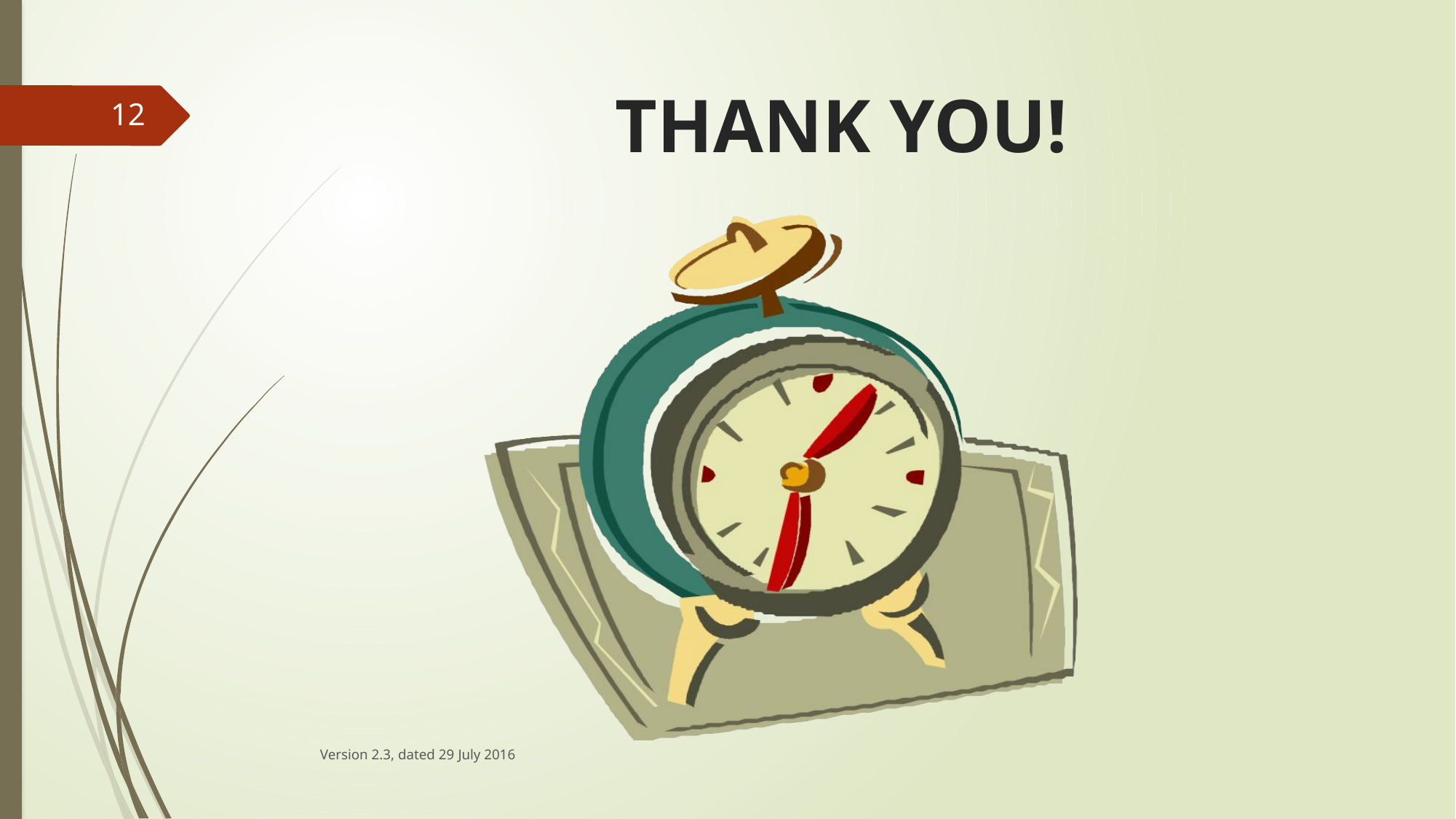

# THANK YOU!
12
Version 2.3, dated 29 July 2016
