# Supplementary material for: The Enhanced Mentor Mother ProgrAm (EMMA) for the prevention of mother-to-child transmission of HIV in Kenya: study protocol for a cluster randomized controlled trial
Source: Trials. 2018 Oct 30;19:594. doi: 10.1186/s13063-018-2975-y (PMC6208066; doi:10.1186/s13063-018-2975-y)
Supplement: Supplementary file 4 — EMMA CRFs (Mother visit data extraction form; mother-infant data extraction form; Mentor-Mother/patient visit record form). (PDF 176 kb) [file 13063_2018_2975_MOESM4_ESM.pdf]

Case Report Forms

For

Protocol RV465 / WRAIR # 2330

KEMRI 0026/3246/BU#H-35152

Version 2.4

26 April, 2017

Implementing the PMTCT standard of care under routine conditions with and without the *E*nhanced *M*entor *M*other Progr*A*m (EMMA): A site-randomized impact evaluation study among maternal and child health clinics supported by the the South Rift Valley PEPFAR Program in Kenya

Short title: **The EMMA study**

Study Conducted by US Military HIV Research Program

Study Supported by

Data Coordinating and Analysis Center, (DCAC), MHRP

Henry M. Jackson Foundation (HJF)

SUMMARY of RV465 CASE REPORT FORMS

Case Report Form	Page No.	Total No.	Visit	Source Doc
1. EMMA Mother Visit Data Extraction Form	4	2		x
2. Mother- Infant Extraction Form	6	2		x
3. EMMA Mentor-Mother/Patient Visit Record Form	8	1		x

Subject ID: |__|__|-|__|__| **Visit Date:** |__|__|-|__|__|__|-|__|__|__|__|

RV465 Case Report Form
General Completion Instructions

(See detailed information in CRF Completion Instruction)

All entries:

- Case Report Forms (CRF) must be completed for every subject who is assigned a subject identification number SUBJECT ID for this study.
- Record the Subject ID in format stated in the protocol, which is 4 digits [first 2 site code, next 2 enrollment number (01-30 per site)]
- Use black or blue ballpoint pen to write entries; d**o <u>not</u> use pencil**.
- Mark with an 'X' in applicable check boxes.
- Avoid using abbreviations unless otherwise specified.

.

Corrections on the CRF:

- **Do <u>not</u> erase entries; do <u>not</u> use correction fluid**
- Draw a single line through the incorrect entry
- Write the correct information close to the original entry; do **<u>not</u>** write over the previous entry.
- Initial and date the corrections. A**<u>ll</u> changes must be initialed and dated by study personnel.**

Shapes/Lines on the CRF:

- ☐ A check box - Mark **all** options that apply following the check boxes
- ◯ A circle field - Mark only **one** that apply among the options
- |__| When brackets are provided for a field, enter **one** character per box.
- |________| A short line with a bar at both ends is a field of a numeric value.
- ________________ A long line after **Other, Specify/Comments** label is a field for long text entry.

Dates on the CRF:

- Dates will be recorded in DDMONYYYY format, e.g., 01JAN2012.
- **DD** is for day and it must be given in 2 digits. (for example: 01, 02, 14, 25 etc…)
- **MMM** is for month and it must be written only the first 3 letters of the month in **English** language as: (JAN, FEB, MAR, APR, MAY, JUN, JUL, AUG, SEP, OCT, NOV, DEC)
- **YYYY** is for year and must be given in 4 digit (for example: 2017)

QC/QA: ________________ **Data Entry: 1st** ________________ **2nd** ________________

This material is the property of WRAIR, and may not be disclosed or used except as authorized in writing by WRAIR.

EMMA MOTHER VISIT DATA EXTRACTION FORM

Subject ID: |__|__|–|__|__| **Visit Date:** |__|__|-|__|__|__|-|__|__|__|__|

EMMA MOTHER VISIT

Nature of Visit: ◯1 Scheduled ◯2 Unscheduled

Note to data extraction team: Fill in once for first ANC visit

PMTCT entry: ◯1 New HIV diagnosis ◯2 HAART clinic

Most recent CD4 result*: |________________| cells/mm³ **Result Date:** |__|__|-|__|__|__|-|__|__|__|__|

Most recent VL result*: |________________| copies/mL **Result Date:** |__|__|-|__|__|__|-|__|__|__|__|

(*If CD4 or Viral Load testing not previously done, please enter N/A for result)*

Date of last menstrual period (LMP): |__|__|-|__|__|__|-|__|__|__|__|

Expected date of child birth: |__|__|-|__|__|__|-|__|__|__|__|

Gestation in weeks: |_________|

Was this patient on ART prior to 1st visit for antenatal care for this pregnancy? ◯1 Yes ◯0 No

Date Started on ARVs: |__|__|-|__|__|__|-|__|__|__|__|

Medications (ordered during this visit)

Medication	Strength	No. of Days Supplied	Medication	Strength	No. of Days Supplied
1.			5.		
2.			6.		
3.			7.		
4.			8.		

What tests were ordered for the patient? (*Mark all that apply*)

- ☐ None
- ☐ Full Hemogram
- ☐ CD4 Count Assay
- ☐ Creatinine
- ☐ HIV Viral Load
- ☐ VDRL
- ☐ ALT (Alanine Aminotransferase)
- ☐ AST (Aspartate Aminotransfrase)
- ☐ Electrolytes
- ☐ Pregnancy Test
- ☐ Sputum for AFB
- ☐ Chest x-ray
- ☐ Other, *specify*: ____________________

CD4 result if ordered at this visit: |________________| cells/mm³

HIV Viral load result if ordered at this visit: |________________| copies/mL

Form Completed by: ____ Date: |__|__|-|__|__|__|-|__|__|__|__|

QC/QA: _______________ **Data Entry: 1st** _______________ **2nd** _______________

This material is the property of WRAIR, and may not be disclosed or used except as authorized in writing by WRAIR.

EMMA MOTHER VISIT DATA EXTRACTION FORM

Subject ID: |__|__|–|__|__| **Visit Date:** |__|__|-|__|__|__|-|__|__|__|__|

EMMA MOTHER VISIT

What referrals were made for the patient? (*Mark all that apply*)

☐ None ☐ Alcohol Counseling/Support groups ☐ Psychosocial Counseling
☐ Disclosure Counseling ☐ Family Planning Services ☐ TB Treatment/DOT Program
☐ Inpatient care/Hospitalization ☐ Nutritional Support ☐ Adherence Counseling
☐ Social Support Services ☐ Mental Health Services ☐ Other, *specify*: ____________________

Was this patient transferred to another facility for future HIV care after this visit?

○ Transferred out? Date: |__|__|-|__|__|__|-|__|__|__|__|

Where: ____________________

○ Died? Date: |__|__|-|__|__|__|-|__|__|__|__|

When is the patient's next appointment? (*Mark appropriate field*)

○$_1$ 1 week ○$_2$ 2 weeks ○$_3$ 1 month ○$_4$ 3 months ○$_5$ 6 months ○$_{88}$ Other, *specify*: ____________

Next Scheduled Appointment, Date: |__|__|-|__|__|__|-|__|__|__|__|

Professionals seen at this visit	Mark 'Yes or No'	Professionals seen at this visit	Mark 'Yes or No'
1. Doctor	○$_1$Yes ○$_0$No	4. Counselor	○$_1$Yes ○$_0$No
2. Clinical Officer	○$_1$Yes ○$_0$No	5. Lab Technician	○$_1$Yes ○$_0$No
3. Nurse	○$_1$Yes ○$_0$No	6. Pharmacist	○$_1$Yes ○$_0$No

Additional Professionals seen, *specify:* ____

____

Form Completed by: ________________________________ Date: |__|__|-|__|__|__|-|__|__|__|__|

QC/QA: ______________ **Data Entry: 1st** ______________ **2nd** ______________

This material is the property of WRAIR, and may not be disclosed or used except as authorized in writing by WRAIR.

MOTHER-INFANT EXTRACTION FORM

Subject ID: |__|__|–|__|__|

MOTHER PROFILE

Date of Birth: |__|__|-|__|__|__|-|__|__|__|__| **Para:** |________| **Gravida:** |________|

Mode of Delivery: ◯ 1 SVD ◯ 2 C-Section

Place of Delivery: ◯ 1 Facility ◯ 2 Home

Mother counselled on feeding options: ◯ 1 Yes ◯ 0 No *Mark below feeding option chosen:*

◯ 1 Exclusive Breastfeeding ◯ 2 Exclusive Replacement Feeding ◯ 3 Mixed

INFANT PROFILE

Date of Birth: |__|__|-|__|__|__|-|__|__|__|__| **Birth Weight (kg):** |________|

Sex: ◯ 1 Male ◯ 2 Female

Alive: ◯ 1 Yes ◯ 0 No **If *No*, date of death:** |__|__|-|__|__|__|-|__|__|__|__|

LABORATORY INFORMATION

Date Sample Collected (DD-MMM-YYYY)	HIV Test Type*	Results**
|__|__|-|__|__|__|-|__|__|__|__|		
|__|__|-|__|__|__|-|__|__|__|__|		
|__|__|-|__|__|__|-|__|__|__|__|		
|__|__|-|__|__|__|-|__|__|__|__|		
|__|__|-|__|__|__|-|__|__|__|__|		
|__|__|-|__|__|__|-|__|__|__|__|		
|__|__|-|__|__|__|-|__|__|__|__|		
|__|__|-|__|__|__|-|__|__|__|__|		

***HIV Test Type: 1. PCR 2. Antibody**

****Results: 0. Negative 1. Positive 2. Indeterminate 3. No Result**

"Ideal timing for testing is at the 6 weeks of age, 6 months of age, 12 months of age, and 18 months of age, however please include all tests completed on the extraction form."

Form Completed by: ______________________________ Date: |__|__|-|__|__|__|-|__|__|__|__|

QC/QA: ______________ **Data Entry: 1st** ______________ **2nd** ______________

This material is the property of WRAIR, and may not be disclosed or used except as authorized in writing by WRAIR.

MOTHER-INFANT EXTRACTION FORM

Subject ID: |__|__| - |__|__|

GROWTH, NUTRITION, AND DEVELOPMENT MONITORING

Visit Date* (DD-MON-YYYY)	Age (wks/ mths)	Weight (Kgs)	Height (cm)	Infant Feeding**	Medication (Indication dose)				TB Assessment Outcome***	Milestones**** Normal (N) Delayed (D) Regressed (R)	Date of Next Appointment (DD-MON-YYYY)
					AZT (mls)	NVP (mls)	CTX (mls)	M/Vit (Y/N)			
(a)	(b)	(c)	(d)	(e)	(f)	(g)	(h)	(i)	(j)	(k)	(l)
|__|__|-|__|__|__|-|__|__|__|__|											|__|__|-|__|__|__|-|__|__|__|__|
|__|__|-|__|__|__|-|__|__|__|__|											|__|__|-|__|__|__|-|__|__|__|__|
|__|__|-|__|__|__|-|__|__|__|__|											|__|__|-|__|__|__|-|__|__|__|__|
|__|__|-|__|__|__|-|__|__|__|__|											|__|__|-|__|__|__|-|__|__|__|__|
|__|__|-|__|__|__|-|__|__|__|__|											|__|__|-|__|__|__|-|__|__|__|__|
|__|__|-|__|__|__|-|__|__|__|__|											|__|__|-|__|__|__|-|__|__|__|__|
|__|__|-|__|__|__|-|__|__|__|__|											|__|__|-|__|__|__|-|__|__|__|__|

*** Visit Dates should correspond to the test dates above.**

****Infant Feeding 1. Exclusive Breastfeeding (EBF) 2. Exclusive Replacement Feeding (ERF) 3. Mixed (BF+RF)**

*****TB Assessment Outcome 1. No signs 2. Presumed TB 3. Confirmed 4. TB Rx 5. Not done (ND)**

******Milestones by Age**

Age Ranges	Milestones
4-6 Weeks	Social Smile
1-3 Months	Head Holding/Control
2-3 Months	Turns toward the origin of sound

5-9 Months	Sitting
7-13 Months	Standing
12-18 Months	Walking
9-24 Months	Talking

Form Completed by: ______________________________ Date: |__|__|-|__|__|__|-|__|__|__|__|

This material is the property of WRAIR, and may not be disclosed or used except as authorized in writing by WRAIR

EMMA Mentor-Mother/Patient Visit Record Form

Subject ID: |__|__|-|__|__| **Visit Date:** |__|__|-|__|__|__|-|__|__|__|__|

1. Age of baby or gestational age (Mark what applies during this visit and circle wks or month)

◯ 1 Gestational age |___________| (wks / months)
◯ 2 Age of Infant/baby |___________| (wks / months)

2. Did Mentor-mother recap all the key clinic processes with the patient/mother? (appointment schedule should guide the discussion, attend to any questions and clarifications.)

◯ 1 Yes
◯ 0 No

3. Was EMMA Mentor-mother guidelines (Adherence, Psychosocial support, Treatment support, Partner involvement) discussed during final interaction with patient/mother?

◯ 1 Yes
◯ 0 No

4. Contact information – Mark all that apply (ensure contact tracing for is updated.)

☐ Full consent for telephonic follow up
☐ Call only
☐ Sms / Text only
☐ No consent for telephonic follow up
☐ Full consent for home follow up
☐ No consent for home follow up

5. Next visit date: |__|__|-|__|__|__|-|__|__|__|__|

6. Text / Sms reminder scheduled on EMMA system based on No. 4 information.

◯ 1 Yes
◯ 0 No

Comments:____

____

Form Completed by: ________________________________ Date: |__|__|-|__|__|__|-|__|__|__|__|

QC/QA: _______________ **Data Entry: 1st** _______________ **2nd** _______________

This material is the property of WRAIR, and may not be disclosed or used except as authorized in writing by WRAIR.
