# Supplementary material for: The Enhanced Mentor Mother ProgrAm (EMMA) for the prevention of mother-to-child transmission of HIV in Kenya: study protocol for a cluster randomized controlled trial
Source: Trials. 2018 Oct 30;19:594. doi: 10.1186/s13063-018-2975-y (PMC6208066; doi:10.1186/s13063-018-2975-y)
Supplement: Supplementary file 5 — Overview of costing analysis. (PPTX 94 kb) [file 13063_2018_2975_MOESM5_ESM.pptx]

## Slide 1
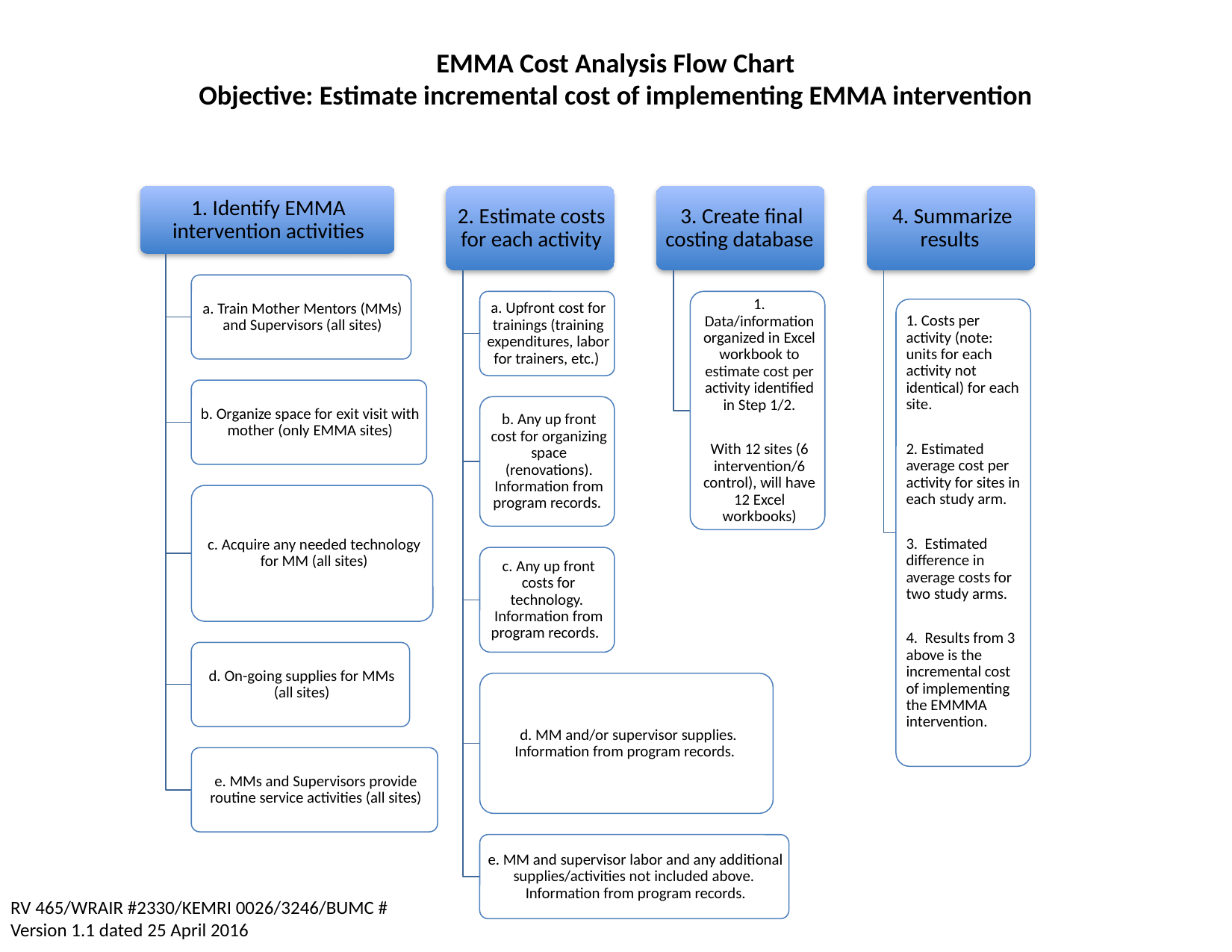

# EMMA Cost Analysis Flow Chart Objective: Estimate incremental cost of implementing EMMA intervention
RV 465/WRAIR #2330/KEMRI 0026/3246/BUMC #
Version 1.1 dated 25 April 2016
